# Can automated content analysis be used to assess and improve the use of evidence in mental health policy? A systematic review

**DOI:** 10.1186/s13643-018-0853-z

**Published:** 2018-11-15

**Authors:** Kristel Alla, Florin Oprescu, Wayne D. Hall, Harvey A. Whiteford, Brian W. Head, Carla S. Meurk

**Affiliations:** 1 School of Public Health, Faculty of Medicine, The University of Queensland, Herston, QLD 4006 Australia; 2 Queensland Centre for Mental Health Research, The Park Centre for Mental Health, Archerfield, QLD 4108 Australia; 3 School of Health and Sport Sciences, The University of the Sunshine Coast, Maroochydore DC, QLD 4558 Australia; 4 Centre for Youth Substance Abuse Research, CYSAR K Floor, Mental Health Centre, Royal Brisbane and Women’s Hospital Campus, The University of Queensland, Herston, QLD 4029 Australia; 5 School of Political Science, The University of Queensland, St Lucia, QLD 4072 Australia

**Keywords:** Automated content analysis, Evidence-informed policy, Research impact, Mental health, Wordscores

## Abstract

**Background:**

This review assesses the utility of applying an automated content analysis method to the field of mental health policy development. We considered the possibility of using the Wordscores algorithm to assess research and policy texts in ways that facilitate the uptake of research into mental health policy.

**Methods:**

The PRISMA framework and the McMaster appraisal tools were used to systematically review and report on the strengths and limitations of the Wordscores algorithm. Nine electronic databases were searched for peer-reviewed journal articles published between 2003 and 2016. Inclusion criteria were (1) articles had to be published in public health, political science, social science or health services disciplines; (2) articles had to be research articles or opinion pieces that used Wordscores; and (3) articles had to discuss both strengths and limitations of using Wordscores for content analysis.

**Results:**

The literature search returned 118 results. Twelve articles met the inclusion criteria. These articles explored a range of policy questions and appraised different aspects of the Wordscores method.

**Discussion:**

Following synthesis of the material, we identified the following as potential strengths of Wordscores: (1) the Wordscores algorithm can be used at all stages of policy development; (2) it is valid and reliable; (3) it can be used to determine the alignment of health policy drafts with research evidence; (4) it enables existing policies to be revised in the light of research; and (5) it can determine whether changes in policy over time were supported by the evidence. Potential limitations identified were (1) decreased accuracy with short documents, (2) words constitute the unit of analysis and (3) expertise is needed to choose ‘reference texts’.

**Conclusions:**

Automated content analysis may be useful in assessing and improving the use of evidence in mental health policies. Wordscores is an automated content analysis option for comparing policy and research texts that could be used by both researchers and policymakers.

**Electronic supplementary material:**

The online version of this article (10.1186/s13643-018-0853-z) contains supplementary material, which is available to authorized users.

## Background

Academics are increasingly expected to inform policy and influence policymakers to produce and implement evidence-based recommendations. This imperative is based on the assumption that evidence-informed policy will improve outcomes and efficiencies [1–3]. Incorporating research evidence into health policy is an increasing focus of research scholarship. The extent to which research is translated into policies can be difficult to appraise given the many, often competing influences on policy decisions [4, 5]. Brownson, Chriqui and Stamatakis [6] have argued that ‘there is a considerable gap between what research shows is effective and the policies that are enacted and enforced’ (p. 1576). Previous work by Katikireddi, Higgins, Bond, Bonell and Macintyre [7], on the formulation of health policy in England, has shown that while some health policy recommendations agree with research evidence, many do not, and some promoted interventions have been shown to be ineffective.

Mental health is one area in which policy development is said to often overlook the research evidence base, resulting in mental health systems that have not reduced the disease burden attributable to mental illness [8, 9]. There have been repeated calls to better incorporate scientific evidence on the most effective interventions into mental health policies and services [10–12]. Zardo, Collie and Livingstone [3] have argued that evidence-informed policy requires tools that facilitate the translation of evidence into effective interventions and policies. Brownson, Chriqui and Stamatakis [6] echoed the need for ‘systematic and evidence-based approaches to policy development’ (p. 1576).

An emergent literature has examined policy and policy processes to account for barriers to research uptake, but the use of research evidence has most often been measured by a qualitative exploration of policymaker perceptions rather than by an examination of the use of research evidence in policy documents [4, 13]. Gibson, Kelvin and Goodyer [4] have suggested that a ‘potential starting point for evaluating direct use of evidence is to examine policy itself, rather than the policy process’ (p. 8). One significant impediment to successful research translation is the large volume of text that needs to be processed when assessing whether research results have been incorporated into policy [14–16]. The dominant method used to examine these texts has been content analysis in which ‘scholars manually code text units and then construct from the existence and frequency of the coded units the occurrence of concepts’ ([17], p. 6). This approach often involves considerable labour and time to create codes and conduct the analysis [18]. Concerns about the reliability of the coding and analysis have prompted researchers to seek alternative methods to reduce inconsistencies and biases arising from human coding [19, 20].

Automated content analysis is a computerised method used to extract meaningful patterns and associations from large textual documents [21, 22]. The method analyses texts in a similar manner to traditional researcher-driven content analysis in that the method of analysis entails a systematic coding and categorisation of text units based on their frequency and co-occurrence [18, 19, 23]. While some would argue that automated coding lacks the capability of capturing the full textual nuances [14], others such as Eriksson and Giacomello [24] remind us that computers only follow researcher instructions. Angus, Rintel and Wiles [22] argue that automated content analysis techniques aim to support rather than replace researchers and that our understanding of how computers can best support research activities is still evolving.

Several automated content analysis tools have emerged in the last decade that differ in analysis techniques and the level of human involvement required. This paper focuses on one method, Wordscores, an algorithm that was developed by Laver, Benoit and Garry [25] in 2003. It can be freely downloaded and used as a plug-in to many widely used statistical software packages (e.g. Stata, R and Java).

Wordscores has been documented as the most popular automated content analysis method in political science, where it has been predominantly used to examine political preferences [26, 27]. Wordscores uses supervised text scaling, which here means that sample texts are classified by experts into predetermined categories which are used on new texts to produce policy estimates [14, 27].

Wordscores has most often been used to analyse policy positions in party manifestos, legislative speeches and policy documents to identify shifts in party ideology on a traditional left-right scale. This includes analysis of policy positions in a wide range of health-related areas, such as economic and social welfare policies. While we are unaware of published examples of this approach being applied to the analysis of mental health policy, it has been used in other health-related policy areas to analyse the influence of the tobacco industry on government policies [28] and in bioethical analyses [29].

Wordscores classifies documents based on word frequencies [14]. The Wordscores algorithm treats words as data and uses a probabilistic technique to score [30] known policy positions expressed in texts provided by the researcher that identify specific policy positions by using word frequencies [28]. Wordscores then maps so-called virgin texts, or texts with unknown positions, to the ‘reference texts’ using weighted averages of the word scores used [28, 31]. This technique treats words purely as data and so disregards semantic information. Wordscores identifies similarities and differences in the patterns of word frequencies between texts. If words scored in a virgin text have unequal relative frequencies to reference texts, this difference is expressed as a difference in position between the two texts.

The aim of this review was to systematically investigate the benefits and limitations of using Wordscores in mental health policy research. Our study specifically asked (1) What types of documents have been analysed using Wordscores and in relation to what research questions? and (2) What are the potential strengths and limitations of using Wordscores to examine mental health research and policy? The analysis considered a range of potential applications for automated content analysis with regard to two groups of stakeholders who have interests in the production of evidence-informed mental health policy, namely researchers and policymakers.

## Methods

The research questions were developed by discussions among all the authors. KA conducted a systematic literature review following the Preferred Reporting Items for Systematic Reviews and Meta-Analyses (PRISMA) framework guidelines for reporting of the article selection criteria and results of systematic reviews [32]. KA carried out database searches for peer-reviewed article abstracts, the application of inclusion criteria and rating of relevancy, article classification and rating and data extraction. FO contributed to search development, article selection, interpretation of the results and cross-verified coding. All authors were involved in the thematic synthesis of findings and the generation of conclusions. This review is not registered with PROSPERO. The PRISMA checklist for this review is provided as an additional file (see Additional file 1: PRISMA checklist).

### Search strategy

The search was conducted between May 2015 and April 2016 using the primary search string: ab(“evidence” AND “research” AND polic*) AND tx(measur*) AND tx(wordscore* OR “word score*”) AND tx(health OR “mental health” OR wellbeing). Electronic databases ProQuest, CINAHL, EMBASE, PubMed, SCOPUS, PsycINFO, Informit, Cochrane Database of Systematic Reviews and Google Scholar were searched by desktop research method, although the precise strategy was adapted to individual databases [16]. The literature search returned a total of 118 results. Sourced article titles and abstracts were screened for relevance to the review aims and scope. Duplicates were rejected. Full texts of chosen articles were downloaded and analysed for inclusion in the final review.

### Eligible studies

**Fig. 1:**
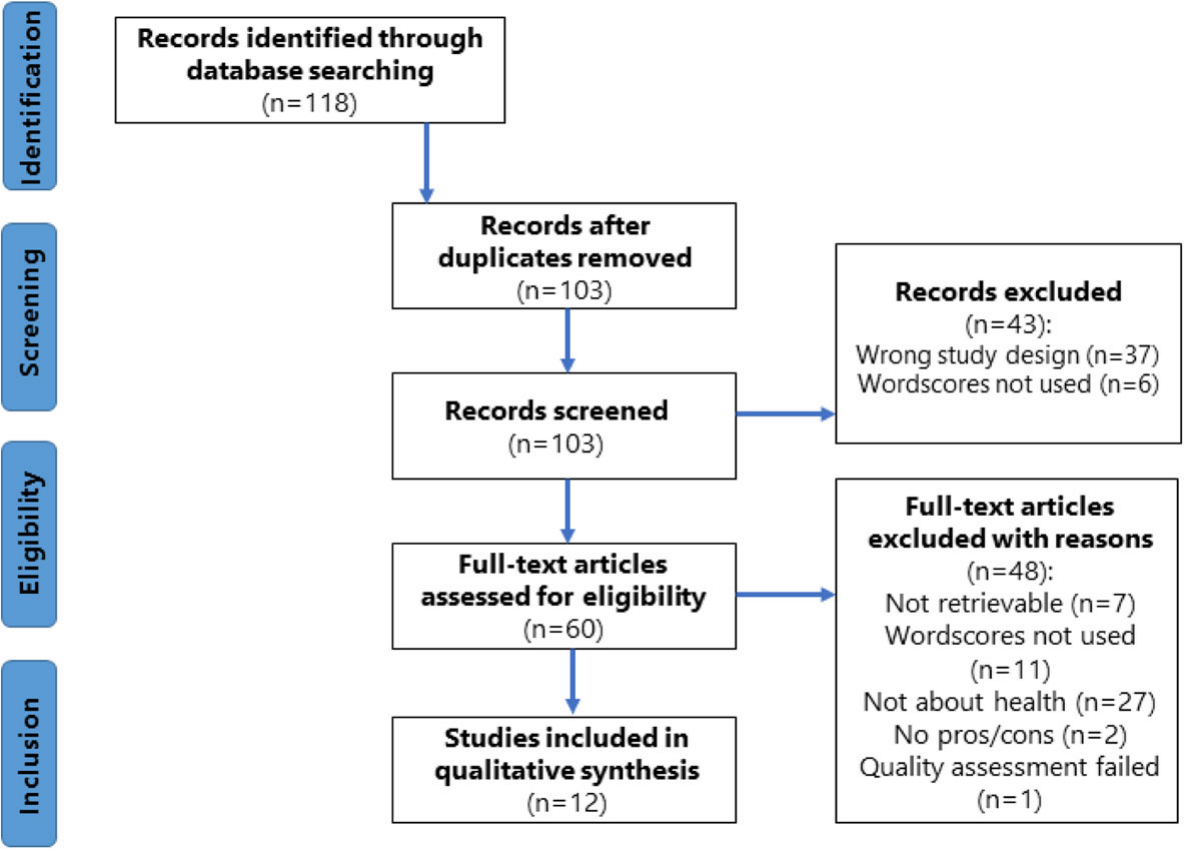
PRISMA flow chart for systematic review method

The literature sample was restricted to peer-reviewed journal articles published between January 2003 and April 2016 because the primary article on the Wordscores method was published in 2003 [25]. Inclusion criteria were (1) articles had to be published in public health, political science, social science or health services disciplines; (2) articles had to be research articles or opinion pieces that used Wordscores; and (3) articles had to discuss both strengths and limitations of using Wordscores for content analysis. Articles that did not meet these criteria were excluded (see Fig. 1 for PRISMA flow chart for the systematic review method).

### Assessment framework

We defined a ‘strength’ as being the potential of the tool to be suitable for assessment of mental health research/policy content. A ‘limitation’ was the potential of the tool to not be suitable for assessment of mental health research/policy content. These qualitative categories were used for data extraction. They involve a degree of subjective assessment that was informed by reported empirical use and assessment, including connotation, of Wordscores in the articles.

### Extraction of data items and attribute appraisal

Subcategories were initially created using a predetermined set of criteria established in a scoping review. The initial list included subcategories: effectiveness, efficiency, usefulness, reliability and expertise required. This list evolved and expanded throughout data analysis, as new subcategories were identified from the literature (Tables 2 and 4 list the final subcategories of strengths/limitations). Each strength/limitation subcategory had to be mentioned in at least two studies to be included in the final analysis. A conclusion on a Wordscores attribute was considered to be made when the article included a declarative statement that included an attribute. Attributes were defined literally (e.g. the statement ‘Wordscores is easy to use’ was coded as ‘strength: easy to use’).

### Critical appraisal of studies

The articles were assessed for quality using the McMaster critical appraisal tools [33]. KA used a systematic approach to extract relevant data from articles and populated a modified McMaster Critical Review Form for each article [34]. The modification of the templates included the addition of four components relevant to the study aims, namely, (1) ‘description of Wordscores’, (2) ‘strengths of Wordscores’, (3) ‘limitations of Wordscores’ and (4) ‘policy relevance’. The templates were used to summarise a critical assessment of the authors’ methods, results, conclusions and potential biases in each article. Each article was critically appraised by one researcher, and quality scores (1–17) were assigned. FO reviewed the templates and quality scores and undertook random cross-verification of the results. Only the articles that had a score over 50% (pass) on the schema were included in this review. One article failed to meet this criterion. Twelve articles met the criteria, and a synthesis of their findings is presented next (see Additional file 2 for an example of a populated McMaster Critical Review Form for review and quality assessment of article by Costa, Gilmore, Peeters, McKee and Stuckler [28]).

## Results

### Documented uses of the Wordscores method

The 12 articles that comprised the sample explored a range of policy questions and appraised different aspects of the Wordscores method. Tabulated summaries are detailed in Table 1. There were nine primary research articles that reported empirical analyses using Wordscores. The sample included three secondary analysis articles (opinion pieces) that discussed the strengths and limitations of Wordscores using health-related policies as case examples [31, 35, 36]. Seven studies concentrated specifically on testing the Wordscores method for its usefulness [25, 29, 30], reliability [25, 28, 30, 37] and validity [25, 30, 37, 38] or its usefulness in identifying policy positions from texts (i.e. on an ideological left-right policy spectrum or aligned to specific lobby group positions). Another two studies determined the strengths and limitations of the Wordscores method [31, 36]. Two studies explored the capabilities of Wordscores in mapping policy changes over time [39, 40]. The study design of all included articles was automated content analysis. Two main types of article were distinguished: (1) studies that aimed to formally assess Wordscores and (2) those that used Wordscores and provided critical comment on its properties. We distinguish between these two types of studies in our summary tables.

**Table 1:** Summary of results

Author	Aim	Wordscores use strengths	Type of document analysed	Methodological considerations	Implications for mental health policy
Baek, Cappella and Bindman* [29]	To explore the usefulness and reliability of automated content analysis of answers to open-ended survey questions from a survey on bioethical issues in genetics research.	Effective, efficient, useful, simple, reliable, high face validity, systematic, versatile, flexible, systematic, consistent, superior to manual coding.	Open-ended cross-sectional survey responses (*n* = 1961) to questions on bioethical issues.	Appropriate, effective for coding purposes and efficient for extracting concepts from large texts. Also effective for analysis of short, informal survey responses. Word usage in reference and target texts needs to be similar for optimal analysis.	Choose reference text samples carefully to ensure comparability.
Baumann, Debus and Müller [41]	To evaluate policy positions on abortion legislation using automated content analysis.	Effective, useful, versatile, flexible, appropriate, strong validity, a promising method that helps inform a rich (nuanced) analysis.	Policy opinion surveys among constituents and speeches on abortion policy in the Irish parliament, 2001–2013, speeches by legislators and advocacy groups.	Effective and reliable method for policy analysis of health-related issues. Suitable for analysis of speeches, debates, policy drafts, advocacy group statements, and constituent surveys.	Consider all stages of the legislative process when selecting a scope of texts for analysis for a richer account.
Bernauer and Bräuninger* [38]	To estimate policy positions of legislators on the left-right scale using Wordscores and to explore links between intra-party faction membership and policy positions.	Effective, reliable, useful, convincing, strong validity, and language-blind (can be used to analyse in any language).	Plenary speeches of legislators (*n* = 453) of the German Bundestag, 2002–2010. Policy positions related to economy and social issues including abortion and euthanasia.	Ensure that ‘virgin’ and reference texts are of similar length and type for optimal comparison.	Comparative research across document types and political actors is recommended. Requires careful selection of reference texts to ensure context requirements are met and analysis is reliable.
Budge and Pennings* [37]	Assesses the Wordscores method for reliability and validity in policy position analysis.	Efficient, simple, systematic, innovative, consistent, promising method.	British party manifestos, 1979–1997.	Promising method for repeated use over time. Results dependent on initial document sets selected for analysis.	Aggregate texts within each time period to create reference sets for pairwise comparisons.
Coffé and Da Roit [39]	To explore changes in party positioning on social and economic issues after a major political event.	Useful, appropriate, reliable.	Party programs for 2006–2008 Italian coalitions: Casa delle Liberta’ and Unione on economic and social policies.	Standard errors accompany each score estimation. No validity issues when rescaling raw score estimates.	Suitable method for analysis of reference texts of varying length and context.
Costa, Gilmore, Peeters, McKee and Stuckler [28]	To determine the influence of the tobacco industry on EU Tobacco Products Directive.	Reliable, simple, objective, innovative, superior to manual coding.	EU Tobacco Products Directive policy drafts, tobacco and health lobby group position papers (*n* = 20).	Requires some prior technical knowledge. Enables visualisations. Clustering effects of virgin text scores: reference text scores tend to be more extreme than in virgin texts.	Efficient for rapid analysis of changes in health policies through different draft stages.
Debus [35]	To explore various methods for analysing policy preferences of political actors.	Robust, reliable, and superior to human coding.	German party programs.	Estimation was left solely to computer algorithms to remove human error. Assumes the systematic use of certain words by policymakers.	Enables the researcher to identify policy or program positions and directions based on automated content analysis.
Hug and Schulz [40]	To assess changes in policy positions over time using various content analysis methods including Wordscores.	Effective, efficient, reliable, strong validity, suitable for retrospective analysis.	Swiss party manifestos, roll call data from the Swiss parliament and voting recommendations, 1991–2003. Range of policy positions including health and social policies.	Wordscores produces most reliable and consistent policy estimates when compared to other content analysis approaches in time series analysis.	Reference texts must have enduring relevance over time period being examined to produce correct measures for policy position changes over time.
Klemmensen, Hobolt and Hansen* [30]	Assesses the Wordscores method for usefulness, reliability and validity in policy position analysis.	Efficient, cost-effective, valid, easy to use, systematic, innovative, flexible, and versatile.	Danish election manifestos from 1945 to 2005 and speeches in parliament.	Supplies time series of policy positions with high face validity. Can be used with Stata/Java. May work best with longer texts.	Can be used for retrospective analysis of policy positions.
Laver, Benoit and Garry* [25]	To explore the usefulness of the Wordscores method for analysing policy positions of legislators.	Effective, efficient, simple, easy to use, quick, reliable, systematic, strong validity, inexpensive, flexible, innovative, versatile, language blind.	Party manifestos and speeches of legislators in the British, Irish and German parliaments on economic and social policies (incl. abortion), expert surveys on policy positions, 1990–1997.	Efficient and rapid method of text analysis with large number of potential applications. Ensure policy assumptions in reference texts are valid prior to analysis. Researcher not required to understand text.	Applicable across languages and contexts but comparisons must be made between texts of similar context and format to ensure validity.
Lowe* [31]	To explore the strengths and weaknesses of Wordscores.	Effective, simple, easy to use, versatile, flexible, and empirically successful.	Other studies conducted using Wordscores method.	Lack of functional and distributional assumptions. Choose reference texts of a similar nature to the texts under investigation.	When using Wordscores, analyse research to select texts suitable for comparison.
Volkens* [36]	To evaluate strengths and weaknesses of three approaches to measuring party policy positions.	Effective, simple, easy to use, quick, reliable, versatile, flexible, promising, high validity, suitable for retrospective analysis, innovative, superior to manual coding, and has useful internal checks and controls.	Other studies conducted on party policy assessment methodologies including Wordscores.	Analysis at specific time points enables the creation of a time line between cause and effect. Focus on text rather than meaning not considered as problematic.	Comparable to expert analysis, but more reliable than manual coding. Wordscores requires skilled researchers to make efficient coding decisions.

*These studies were formal evaluations of Wordscores

The types of documents analysed using Wordscores included policy drafts [28], adopted policies [28], party manifestos [25, 30, 35, 37, 39, 40], plenary speeches [25, 30, 38], transcripts of parliamentary debates [25], position papers [28], roll call data [40], voting recommendations [40] and cross-sectional survey responses [25, 29, 41]. Ten papers reported analyses of multiple types of documents. Automated content analysis was used to assess policies that were directly health-related in six studies [25, 28, 29, 38, 40, 41]. These included studies of the tobacco industry [28], abortion [25, 38, 41] and genetics [29]. Social policies [25, 38, 40] and economic policies [25, 38, 39] were analysed using the automated content method in four studies. Wordscores was used for retrospective analysis of policy positions in three studies [30, 37, 40]. The studies were conducted exclusively in Europe (Germany, England, Ireland and Switzerland) and included three cross-national studies [25, 28, 36].

### Documented strengths of the Wordscores method

Nine studies documented that Wordscores effectively extracted policy positions from large texts [25, 28, 29, 31, 36–38, 40, 41]. These studies confirmed that the technique was effective at consistently generating and comparing policy positions [25, 28, 29, 37, 40]. Baek, Cappella and Bindman [29] advised that the method could be used to establish the character of whole texts. Bernauer and Bräuninger [38] concurred that Wordscores can be used to accurately reflect the perceived positions of individuals and groups alike. Table 2 summarises documented strengths in individual studies.

**Table 2:** Summary of reported strengths in included studies

Study	Ease of use					Versatility				Resource efficiency		Reliability and validity					
	Effective	Simple to use	Easy to use	Quick	Language blind	Versatile	Range of applications	Flexible	Useful	Efficient	Cost-effective	Reliable	Good with large texts	Systematic	Inbuilt cross-validation for reliability	High face validity	Equal or better than manual coding
Baek, Cappella and Bindman* [29]	☑	☑	☑	☑		☑	☑	☑	☑	☑		☑	☑	☑		☑	☑
Baumann, Debus and Müller [41]	☑					☑			☑			☑	☑				
Bernauer and Bräuninger* [38]	☑				☑				☑			☑	☑			☑	
Budge and Pennings* [37]	☑	☑		☑			☑			☑			☑	☑			
Coffé and Da Roit [39]	☑								☑								
Costa, Gilmore, Peeters, McKee and Stuckler [28]	☑											☑	☑				☑
Debus [35]	☑	☑					☑										☑
Hug and Schulz [40]	☑				☑		☑			☑		☑	☑		☑		☑
Klemmensen, Hobolt and Hansen* [30]		☑	☑		☑	☑	☑	☑		☑	☑	☑	☑	☑	☑	☑	☑
Laver, Benoit and Garry* [25]	☑	☑	☑	☑	☑	☑	☑	☑	☑	☑	☑	☑	☑	☑	☑	☑	☑
Lowe* [31]	☑	☑	☑			☑							☑				
Volkens* [36]	☑	☑	☑	☑		☑		☑	☑	☑	☑	☑	☑		☑	☑	☑

*These studies were formal evaluations of Wordscores

#### Ease of use

Wordscores was described as an ‘effortless’ [25], simple [25, 29–31, 36, 37] and quick method to use [25, 29, 37] that analysed texts within a few seconds of turnaround time [25, 36]. An important strength was that it calculated policy positions using computer algorithms [35] integrated with a range of publicly available statistical analysis software [25, 29, 30]. From the perspective of researchers, the automated content analysis was easy to access and use. The computationally straightforward technique [31, 35] had the benefit that the analyst did not need to understand the meaning of the text that was being coded [25, 30, 38, 40]. The studies concluded that this ‘language-blind’ text coding technique [25, 30, 38, 40] can be applied to texts in any language, including those the researcher does not speak [25]. In this regard, Wordscores can greatly simplify the task of content analysis by comparison to traditional content analysis methods.

#### Versatility

The authors identified Wordscores as a useful [25, 29, 36, 38, 39, 41] and versatile [25, 29–31, 36, 41] content analysis method that allowed a high degree of flexibility in its application [25, 29, 30, 36]. Klemmensen, Hobolt and Hansen [30] (p. 754), for example, stated that Wordscores was ‘more flexible than any other method for estimating policy positions’. Studies found the technique agreed with expert views when applied to a range of complex topics [25, 29, 37, 40] that included cross-national analysis and different types of political actors. Debus [35] appreciated the ‘policy blindness’ of Wordscores meaning its lack of discrimination between policy content and issue salience.

Baek, Cappella and Bindman [29] found Wordscores useful in analysing texts of greatly varying length that had been formulated by a broad range of actors and when texts varied in tone from neutral to advocacy pieces. Studies confirmed that the technique was reliable when analysing texts of different formats: policy documents [28], party programs [25, 30, 35, 37, 39, 40], agreements, debates and speeches [25, 30, 38], position papers [28], roll call data [40], voting recommendations [40] and cross-sectional survey responses [25, 29, 41].

Another methodological advantage of Wordscores was its ability to provide data on changes in a series of policy positions over time using a method of high face validity [25, 29, 30, 36, 38]. For example, in their analysis of Danish party manifestos and government speeches over a 50-year period, Klemmensen, Hobolt and Hansen [30] confirmed that Wordscores accurately traced dramatic policy moves over a series of elections.

#### Reliability and validity

Researchers appreciated the inbuilt uncertainty estimates of Wordscores in producing content analysis results that were reliable [25, 28, 29, 36, 38–41] and systematic [25, 29, 30, 37] with strong claims for validity [25, 29, 30, 36, 38]. Several studies found Wordscores as effective as, or superior to, manual coding methods [25, 28, 29, 35, 36, 40] because it reduced human error [30, 35]. The cross-validation mechanism [36] of computing confidence intervals for comparisons of reference and virgin texts was a frequently mentioned advantage of the Wordscores method over other methods [25, 30, 36, 40]. This enabled the researchers to assess whether differences between the positions were significant or could be attributed to measurement error [25].

Six studies including four formal evaluation studies compared Wordscores analysis of texts with an expert assessment using Comparative Manifesto Project data to assess the reliability of Wordscores. The Comparative Manifesto Project [42] was a content analysis project in political science in which expert coders undertook content analysis of over 1000 policy texts, and their coding reliability was assessed. Common validity tests to assess Wordscores compared to other methods included concurrent validity and comparative predictive validity. Only one study used Krippendorff’s alpha [29] as a reliability measure to test Wordscores. Table 3 provides an overview of validity and reliability testing as well as the risk of bias assessments.

**Table 3:** Summary of reliability and validity measures used in included studies

Author	Study design	Reliability and validity measures	Bias
Baek, Cappella and Bindman* [29]	Formal evaluation of Wordscores. Good description of methods. Testing of two automated content analytic methods to assess validity in comparison to manual coding. Specified method of study as ‘testing’.	Completed reliability and validity tests. Used Krippendorff’s alpha (.61 using 7% of reference tests and > .70 using 50% of reference tests), concurrent validity and comparative predictive validity tests.	Only two methods tested for reliability including the method of affective intonation created by authors of this article. This may potentially mean favourable results are expected for this method. Low risk of bias.
Baumann, Debus and Müller [41]	Used Wordscores without a formal evaluation. Some study design issues. Lack of background literature for Wordscores. Descriptive analysis without references to statistical significance. Results tabulated.	None reported.	No limitations to study design or using Wordscores mentioned. Unclear risk of bias.
Bernauer and Bräuninger* [38]	Formal evaluation of Wordscores. Good description of the study design. Descriptive statistics, results tabulated.	Used expert scoring for reliability comparison with Wordscores. Compared results of validity tests. Established strong face validity for Wordscores.	Good discussion of strengths and limitations of study design. Low risk of bias.
Budge and Pennings* [37]	Formal evaluation of Wordscores. Focus is a comparative evaluation of methods. Descriptive statistics, results tabulated.	Used the Comparative Manifesto Project and expert scoring for reliability comparison with Wordscores results. Emphasis on validity and reliability testing. Established some reliability issues for analysing policy positions using Wordscores.	Article is part of a debate series. Starts with premise that computerised content analysis does not work and continues to build a case against the Wordscores. High risk of bias.
Coffé and Da Roit [39]	Used Wordscores without a formal evaluation. Good description of the study design. Descriptive statistics, results tabulated.	Used the Comparative Manifesto Project for reliability comparison with Wordscores results.	No limitations to study design mentioned. Unclear bias.
Costa, Gilmore, Peeters, McKee and Stuckler [28]	Used Wordscores without a formal evaluation as its aim. Study design described in detail and advised as quantitative automated content analysis. Descriptive statistics, results tabulated.	Used various reference texts to test the reliability of Wordscores. No expert comparisons or validity tests performed.	Accounted for potential issues with reliability and validity for Wordscores. Wordscores thoroughly assessed for both strengths and limitations. Low risk of bias.
Debus [35]	Editorial. Good description of different methods of content analysis, including Wordscores. Good overview of strengths and benefits of Wordscores compared to other content analysis methods. No data sets analysed. No result tables.	None reported.	Editorial for a special issue dedicated to content analysis methods including automated content analysis and therefore potential bias in favour of automated content analysis methods. High risk of bias.
Hug and Schulz [40]	Used Wordscores without a formal evaluation as its aim. Good description of methods. Several analyses conducted with different reference texts to test method. Descriptive statistics, results tabulated. Measures to statistically significant levels.	Completed reliability and validity tests. Used expert data and the Comparative Manifesto Project for reliability comparison with Wordscores, accounted for limitations in all methods.	Potential impacts on reliability and validity assessed. Wordscores thoroughly assessed for both strengths and limitations. Low risk of bias.
Klemmensen, Hobolt and Hansen* [30]	Formal evaluation of Wordscores. Good description of methods. Several analyses conducted with different reference texts to test method. Descriptive statistics, results tabulated. Measures to statistically significant levels. Good quality analysis.	Completed reliability and validity tests. Used expert data, the Comparative Manifesto Project for reliability comparison with Wordscores and another automated method (Spearman’s rho used for correspondence analysis).	Potential impacts on reliability and validity assessed. Wordscores thoroughly assessed for both strengths and limitations. Low risk of bias.
Laver, Benoit and Garry* [25]	Formal evaluation of Wordscores. Stated as study design cross-validation of different methods to validate policy estimates. Used both English and non-English speaking texts for cross-validation. Good description of methods.	Completed reliability and validity tests using expert data, the Comparative Manifesto Project and Wordscores for comparison.	Potential impacts on reliability and validity assessed. Wordscores thoroughly assessed for both strengths and limitations. These are authors of the Wordscores method. Low risk of bias.
Lowe* [31]	Formal evaluation of Wordscores. Good overview of the mechanics of how Wordscores works. Focuses on processes which Wordscores algorithm uses for score estimation and its reliability.	Core focus is on reliability testing of Wordscores.	Starts with a hypothesis that there are issues with Wordscores and continues to build a case against the method. High risk of bias.
Volkens* [36]	Formal evaluation of Wordscores. Tabulated overviews of the strengths and the weaknesses of three methods evaluated.	Used expert data, the Comparative Manifesto Project for reliability comparison with Wordscores and another automated method (CACA). Good overview of method reliability and validity in comparison.	Potential impacts on reliability and validity assessed. Wordscores thoroughly assessed for both strengths and limitations. Low risk of bias.

#### Resource efficiency

Several studies found that Wordscores was as effective as, or superior to, manual coding methods and so cost-effective [25, 30, 36] because it made more efficient use of expensive personnel resources [25, 29, 36]. Available literature suggests that Wordscores is an efficient technique for text content analysis [25, 29, 30, 36, 37, 40]. For example, Klemmensen, Hobolt and Hansen [30] (p. 754), stated that Wordscores ‘offers a cheap, efficient and language-blind technique for extracting policy positions from political texts’.

### Documented limitations of the Wordscores method

None of the studies reviewed reported that Wordscores failed to provide reliable results when applied to complex policies. Nevertheless, some highlighted important limitations of using Wordscores in policy analysis (see Table 4).

**Table 4:** Summary of reported limitations in included studies

Study	Text length	Relationship between words, meaning and context		Expertise needed to inform the choice of reference texts				
	Less accurate with short texts	Word focussed	Applicability to complex contexts	Reference texts must fulfil certain conditions	Scores require rescaling	Potential for transformation errors	Requires researcher interference and skills	Score quality of reference texts
Baek, Cappella and Bindman* [29]		☑	☑	☑				
Baumann, Debus and Müller [41]		☑						
Bernauer and Bräuninger* [38]		☑		☑				
Budge and Pennings* [37]		☑			☑	☑		☑
Coffé and Da Roit [39]		☑						
Costa, Gilmore, Peeters, McKee and Stuckler [28]	☑	☑		☑	☑	☑	☑	☑
Debus [35]		☑		☑			☑	
Hug and Schulz [40]	☑			☑				
Klemmensen, Hobolt and Hansen* [30]	☑	☑		☑		☑	☑	☑
Laver, Benoit and Garry* [25]	☑			☑			☑	☑
Lowe* [31]		☑		☑	☑			☑
Volkens* [36]	☑	☑	☑	☑			☑	

*These studies were formal evaluations of Wordscores

#### Document length

One of the most discussed limitations of Wordscores was its decreased accuracy when analysing short documents. Many studies highlighted that the method was better suited to the analysis of lengthy documents [25, 28, 30, 36, 40].

#### Relationship between words, meaning and context

Another common criticism of Wordscores was its central focus on words as the unit of analysis. Several authors argued that only analysing word frequency produced a one-dimensional analysis [28–30, 35, 39] and prevented an understanding based on the nuanced ways that words are used and the context in which policy texts are produced [31]. Costa, Gilmore, Peeters, McKee and Stuckler [28] expressed reservations about the extent to which words alone could accurately reflect policy positions and argued that Wordscores could only provide comparisons of the policy positions in the studied text with that of the reference texts. Coffé and Da Roit [39] questioned the assumption that actors consciously choose words that expressed their policy positions. They argued that Wordscores relies on the premise that word choices reflect the ideology of the person/party that addresses them, whereas word choices of actors may not necessarily be deliberate [39]. This raises questions as to the extent to which Wordscores analyses are valid. For these reasons, some authors were unsure whether Wordscores could be applicable to more complex policy contexts [29, 36].

#### Expertise needed to inform the choice of reference text

Many authors highlighted the fact that the validity of Wordscores analyses depends on the reference texts that are selected by the investigator and used as the basis for appraising virgin texts. This required expert knowledge of the policy area being analysed, contrary to the claims that use of Wordscores does not require expert knowledge. Specifically, some authors argued that reference texts provide better results when they (1) have the same purpose and context (lexicon) as the texts under analysis [25, 29, 30, 35, 40], (2) contain both ‘extreme’ and centre positions [25, 28, 30] and (3) are heterogeneous in terms of word structure and frequency [28, 30, 31, 35]. According to Debus [35], ensuring the homogeneity of reference texts in language and ideological background (i.e. using documents from the same country) “decreases the chances of cross-national, comparative analysis of policy positions, e.g. the analysis of similar or deviating positions of specific policy area positions of parties belonging to similar ideological ‘families’” (p. 291). It is therefore important that the researcher labels the keywords correctly to enable comparisons [35]. In summary, Wordscores requires researcher expertise and technical skill but less than some other content analysis methods according to Laver, Benoit and Garry [25].

## Discussion

**Fig. 2:**
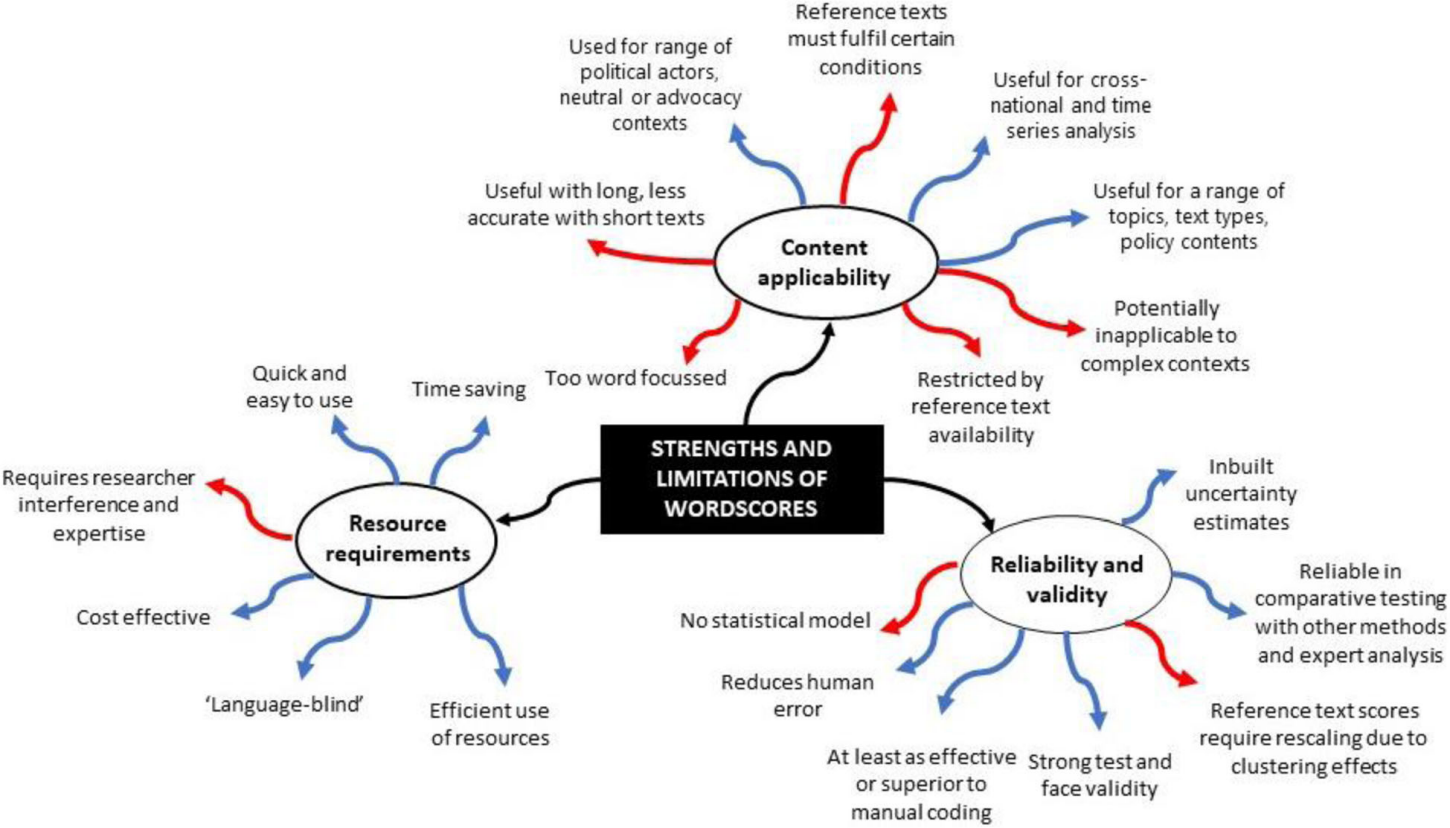
Summary of strengths and limitations of Wordscores

This review systematically analysed 12 articles on policy that used the Wordscores method. The review found no documented uses of Wordscores in mental health policy analysis, but it has been used in health-related policy analysis, mostly in European countries. Authors who have used Wordscores report it has several strengths that make this a promising tool for investigating various stages of policy development relating to mental health, including its use in understanding the diffusion of research innovation into policy throughout the policymaking process (Fig. 2).

### Usefulness in understanding and developing evidence-informed policy

**Fig. 3:**
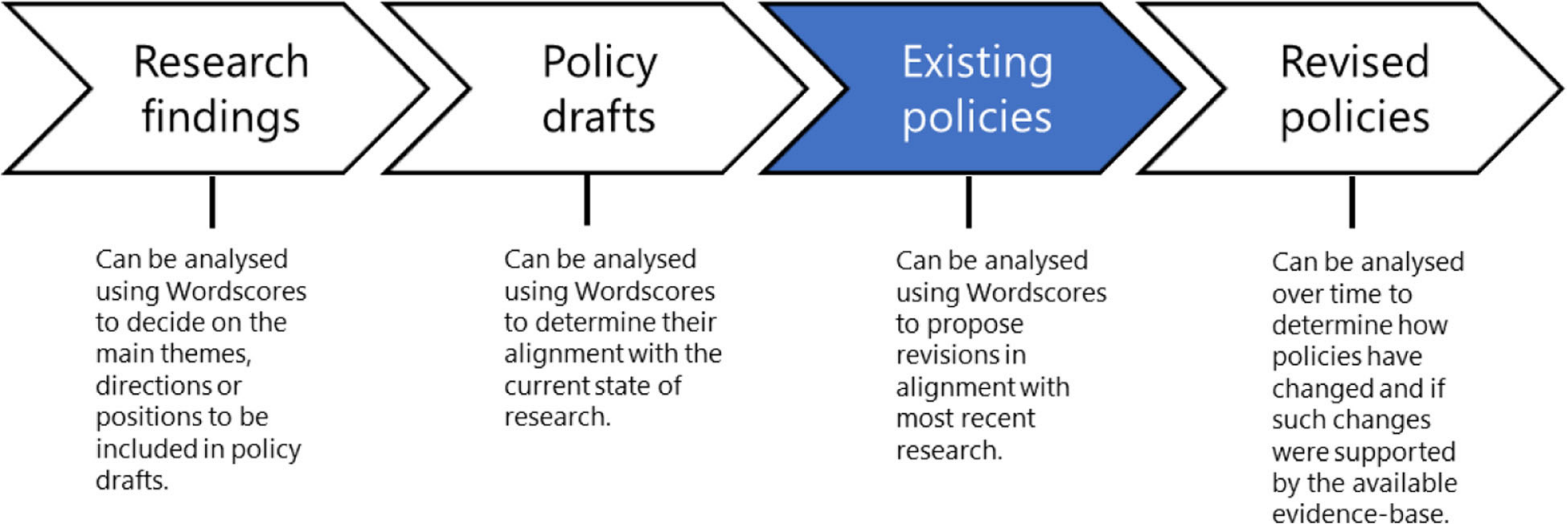
Supporting evidence-based policy development processes using Wordscores

While Wordscores was initially developed for the comparative analysis of policy positions, it has the potential to create evidence reference positions from available texts and to use reference texts to analyse policy drafts, agendas and legislation. Such analyses could be used for academic purposes, i.e. to improve our understanding of the way evidence circulates through policy or as a basis for proactively intervening in the policy development process. For example, the analysis of policy positions at successive time points makes it possible to monitor how evidence is used over time (i.e. during the revisions of a policy). The Wordscores method could thus potentially aid ongoing policy evaluation with its results fed back into the policy development cycle in an adaptive way (see Fig. 3).

What we propose for the future is an innovative use of Wordscores, based on the analysis of ‘research positions’ as opposed to ‘policy positions’. Furthermore, this method could be used to analyse both policy and research to highlight both evidence gaps in policies and policy-focussed research gaps. By improving the efficiency of combined policy and research analysis, Wordscores could provide a useful tool for both academics and policy analysts to better understand the role of research evidence in policy formulation.

Stakeholders who may benefit from automated content analysis could be administrators and other policymakers involved in policy analysis. It could also include researchers who want to understand and/or enhance the use of evidence in policy. In making these suggestions, we note that these uses of automated content analysis tools, like Wordscores, for policy development remain untested and thus would require further evaluation.

### Diffusion of mental health research evidence into policy

Social constructivist theories of research utilisation highlight the way in which knowledge is co-created by researchers and users [43, 44]. In this view, knowledge undergoes a continual reshaping that produces new meanings [45]. Policymakers often face competing knowledge claims from scientific researchers, policy entrepreneurs and politicians which they must weigh when using research to formulate specific policies [45]. It could be argued that the applications of Wordscores we have proposed neglect the complex socio-political reality in which policy is made. However, while there may not always be a direct pathway from evidence to policy, our approach may still facilitate knowledge flows and create opportunities to develop evidence-informed policies.

The use of efficient automated content analysis methods such as Wordscores could support policy development by allowing more direct, efficient and effective ways to synthesise and disseminate evidence. At the same time, the Wordscores method can be utilised to measure the diffusion of mental health research into policy because it can provide sequential, repeated and longitudinal measures from draft to implemented policy to revised policy. Research findings on a particular mental health topic could be analysed using Wordscores to identify the main themes, directions or positions that have been included in successive policy drafts. Policy drafts could be analysed using Wordscores to determine their alignment with the current research; existing policies could be analysed using Wordscores to better align policy with the most recent research; and revised policies could be analysed over time to determine how they have changed and if such changes are supported by evidence.

### Limitations of Wordscores

The strengths of Wordscores indicate that it can automate some aspects of policy analysis in ways that require limited expert knowledge. However, effective use of the method depends crucially on the careful choice of reference texts. Limitations relating to the analysis of short texts are of little concern because many health research and policy documents are lengthy, and short documents can be easily analysed using traditional methods.

Wordscores may be most appropriate as an initial form of analysis if it is understood that as an automated method it may not capture the entire meaning intended by authors. Other automated content analysis methods (e.g. Leximancer) that use ‘concepts’ as the unit of measurement rather than words can add a more complex layer of information.

### Study limitations

This review was restricted to English-language studies. There may have been studies conducted on Wordscores in other languages and published in grey literature that were relevant. The assessment framework was bespoke and so has not been tested or verified by other authors. As outlined in the ‘Methods’ section, we used a systematic approach to extract data and assign it into qualitative categories. However, as with any qualitative approach, this involved a degree of subjectivity, and different methods of data collection may have produced a different outcome. However, in most cases, text analysed was taken literally, i.e. if an author stated that Wordscores was ‘easy to use’, then we created a code ‘easy to use’. The majority of data collection, analysis and synthesis was carried out by the lead author with the second author contributing to search development, article selection, interpretation of the results and cross-verification of coding.

This study was limited by the small pool of publications relevant to our research questions. Few studies were relevant to health policy, none to mental health and only seven studies provided formal evaluations of Wordscores. Reporting of strengths and limitations was also variable, with some studies dedicating more space to describing the strengths and limitations of Wordscores than others. Risk of bias assessment was limited because few studies reported bias and conflicts of interest. This probably reflects differences in the conventions and norms of policy studies in comparison to health and medical research fields.

Authors self-selected to use Wordscores in studies and therefore may have been inclined to be less critical of automated content analysis methods. Publication bias is a consideration, given that researchers may not proceed to publication if they deem the method unsuitable for their purposes. Additionally, the authors of these studies are potential experts in automated content analysis, and thus, their assessments of Wordscores that was ‘easy to use’ may be true of someone with their level of expertise rather than researchers in general. We did not assess the level of expertise of the study authors.

We also acknowledge that there are influences on policy decisions that may not be detectable in policy documents. Consequently, automated content analysis tools comprise only one means of interrogating questions about how research influences policy. Other methods for assessment of policy processes should be used in tandem.

### Future research

The articles we reviewed highlighted the importance of selecting suitable reference policy texts to a successful analysis. How relevant ‘research position’ texts might be identified and selected in mental health should be the subject of future research. Another issue for future research on evidence-based health policies would be to explore and compare different automated content analysis methods in addition to Wordscores.

There is insufficient evidence to advocate for the application of Wordscores as we have described it. Rather, we propose that these innovative applications of this technology, and other automated content analysis packages, be further investigated. As demonstrated above, Wordscores has both strengths and limitations. However, the review indicates that it is feasible to further explore the efficacy of this, and similar methods, and their potential applications in an area, such as mental health, where this approach has not previously been used. Firstly, the review provides precedents for the use of this approach in policy studies in ways that should be relevant to mental health policy analysis. Secondly, we believe it is possible to transfer a method used to examine policy positions and use it for a comparative analysis of policy and research evidence. The next step is to evaluate the utility of this approach through empirical testing using case studies.

## Conclusion

Automated content analysis technologies are continuously being developed. While we have focussed on Wordscores in this study, automated content analysis may assist understanding and facilitating evidence-informed policy more broadly. Automated content analysis provides potential analytical tools that could be utilised at various stages of policy development to examine, and facilitate, the diffusion of research (innovation) into policy. Automated approaches, such as Wordscores, cannot and should not replace expert understanding in policy analysis. Automated content analysis is a complementary method that can, when cross-validated using other methods, provide valid data to assist and support expert understanding. By automating some stages of the analysis process, researcher time can be freed up and the research process expedited compared to human coding. This makes it more feasible to follow policy throughout its life cycle. The use of Wordscores, in particular, allows the creation of reference positions and directions based on available evidence to analyse policy drafts, agendas and published legislation to assess whether changes are in line with available research findings. Further investigation of other automated content analysis tools is warranted. Research using automated content analysis should be encouraged and supported in mental healthcare where there is a critical need for strategic research diffusion into policy at all levels.

## Additional files

Additional file 1: PRISMA checklist. (DOC 65 kb)

Additional file 2: Modified McMaster appraisal template example. (DOC 90 kb)
