# Supplementary material for: Can automated content analysis be used to assess and improve the use of evidence in mental health policy? A systematic review
Source: Syst Rev. 2018 Nov 15;7:194. doi: 10.1186/s13643-018-0853-z (PMC6238396; doi:10.1186/s13643-018-0853-z)
Supplement: Supplementary file 2 — Modified McMaster appraisal template example. (DOC 90 kb) [file 13643_2018_853_MOESM2_ESM.doc]

# **Additional file 1. Modified McMaster appraisal template example**

# **Critical Review Form – Quantitative Studies**

**©Law, M., Stewart, D., Pollock, N., Letts, L., Bosch, J., & Westmorland, M. 1998**

**McMaster University**

******** score yes=1, no=0.

CITATION: 13 /17

| Costa H, Gilmore AB, Peeters S, McKee M, Stuckler D: **Quantifying the influence of the tobacco industry on EU governance: automated content analysis of the EU Tobacco Products Directive.** *Tob Control* 2014, **23:**473-478. doi:10.1136/tobaccocontrol-2014-051822 |
| --- |

**Comments**

# Study Scope

| **STUDY** **SCOPE**: | **Study scope**   - EU policy and lobby group tobacco policy related documents analysed and compared for policy positions using word frequencies to map proximity of policy development from proposal to adoption. |
| --- | --- |

# Policy Relevance

| **POLICIES:**  **Were policies described in detail?**  Yes  No  Not addressed | **Provide a short description of the policies and policy approaches used in study (focus, which policy stage was in focus – draft, final legislation, setting). Were any theoretical policy frameworks mentioned? Were policies health-related?**   - EU tobacco policy drafts and adopted legislation in focus and described in sufficient detail. - No theoretical policy frameworks mentioned.   **Relationship to health policies**   - Directly health-related: tobacco legislation in focus that regulated tobacco sales to regulate negative tobacco use impacts on population health. Policy positions extracted from tobacco legislation compared to health NGO positions. |
| --- | --- |

# Purpose

| **STUDY** **PURPOSE**:  **Was the purpose stated clearly?**  Yes  No | **Outline the purpose of the study. How does the study apply to health-related discipline and/or your research question**?   - To determine the influence of the tobacco industry on EU governance applying automated content analysis to the example of the EU Tobacco Products Directive adoption process. |
| --- | --- |

# Definitions

| **DEFINITIONS**:  **Was automated content analysis method explicitly defined?**  Yes  No  **KEY TERMS:**  **What was the terminology used for the method?**  text mining technique  automated content analysis  computerised content analysis  computer-assisted content analysis  automatic content analysis  quantitative content analysis | **Definitions for the automated content analysis method:**   - No definitions proposed for automated content analysis.   **Key terms used for the automated content analysis method:**   - Quantitative text mining technique, automated content analysis (technique). |
| --- | --- |

# Justification and Literature Review

| **LITERATURE:**  **Was the relevant background literature** **reviewed**?  Yes  No | **Describe the justification of the need for this study**.   - Justified by the need to establish whether the tobacco industry investments into lobbying impact the European Union (EU) tobacco policy. - Background given for the European Union (EU) Tobacco Products Directive (TPD) development that regulated the manufacture, marketing and sale of tobacco products, as well as for the pro-tobacco lobbying efforts by lobby groups. Background introduced in terms of relevant information to lobbying efforts and policy development, however scientific research evidence was not incorporated. |
| --- | --- |

# Study Design and Bias

| **DESIGN:**  randomized (RCT)  cohort  single case design  before and after  case-control  cross-sectional  case study  other or unsure  **Was the study design explicitly defined**?  Yes  No | **Describe the study design. Was the design appropriate for the study question? (e.g. for knowledge level about this issue, outcomes, ethical issues etc**.)   - The aim was not to evaluate the effectiveness of Wordscores methodology, this was merely used as a method to analyse data. - Policy positions of 18 stakeholders were explored based on text submissions to EU consultations that included tobacco industry and public health NGO lobby groups and tobacco retailers. - Wordscores (STATAV.13.0).was used to calculate word frequencies (scale from 0–tobacco industry to 1–public health organisations) and to track changes in the TPD 2010 consultation document, its 2012 final proposal and in 2014 adopted legislation. Hypothesized that the tobacco industry uses economy statements more often than health NGOs. - Martin-Vanberg transformation used to ensure comparability of virgin and reference texts using extreme positions. - Study design described in sufficient detail and advised as quantitative automated content analysis.   **Specify any biases that may have been operating and the direction of their influence on the results**.   - Used various reference texts to test reliability of Wordscores. No expert comparisons or validity tests performed. Accounted for potential issues with reliability and validity for Wordscores. Wordscores thoroughly assessed for both strengths and limitations. Low risk of bias. |
| --- | --- |

# Description of WORDSCORES

| **DESCRIPTION OF WORDSCORES**  **Was the Wordscores method explained?**  Yes  No  **KEY TERMS used:**  Wordscores scaling algorithm  Wordscores approach  Wordscores technique  **Tools indicated:**  JFreq  STATAV  Martin-Vanberg transformation | **Was the Wordscores method described in sufficient detail and what importance was given to various aspects**?)   - Clear and concise overview of the Wordscores method was provided, including information and overview of what the method is being used for in political science discipline. - A sufficient description was provided on how the raw data was processed prior to analysis (i.e. words reduced to roots, symbols and numbers excluded). - The Wordscores frequency algorithm was presented. - Coding procedure was described and substantiated. - Wordscores were rescaled using the Martin-Vanberg (MV) transformation to facilitate comparability. |
| --- | --- |

# Strengths of Method

| **STRENGTHS:**  **Were the Wordscores method strengths explained?**  Yes  No  **Strengths mentioned:**  Effective (coding, large texts)  Efficient (coding, large texts)  Simple  Easy to use  Quick  Reliable  Systematic  Objective  Potentially powerful  High face validity  Inexpensive/cost-effective  Not labour intensive  Language blind  Enables retrospective analysis  Flexible  Innovative (or novel)  Versatile (incl. used with Strata/Java)  No prior knowledge of text required for use  Gives measure of uncertainty /w scores  Superior to manual coding/reduces human error  Consistent  Useful  Other  **What wording was used was strengths?**  Strengths  Advantages  Merits  Pros  Other  Described only | **Strengths (what were considered and characteristics; how many?)**   - Increased objectivity and reliability of analysis compared to manual coding processes. - Increased reliability provided by automated rather than human coding of texts. - Effectively established and compared policy positions providing visual representation. - Established that Wordscores enabled to show position changes over time. - Parallel testing conducted to ensure accuracy of results. - Effective method to improve objectivity of content analysis. - Simple and easy to use method to visually map, quantify and communicate policy positions. |
| --- | --- |

# Limitations of Method

| **LIMITATIONS:**  **Were the Wordscores method limitations explained?**  Yes  No  **Which limitations were mentioned?**  Long documents are more reliable  Texts of similar nature (Reference texts must fulfil certain conditions)  Texts with ‘extremes’  Word focussed  Reference texts must differ  Score quality of reference texts  Transformation errors  Clustering effects  Inapplicable to complex contexts  Indistinguishable degrees of comparability  No word nuances  Scores require rescaling  No statistical model  Use depends on reference texts  Requires researcher interference and skills  Language restrictions  Other  **What wording was used for limitations?**  Limitations  Disadvantages  Shortcomings  Cons  Drawbacks  Other  Described | **Limitations (what were considered and characteristics; how many?)**   - Method lies on the premise that word choices reflect the ideology of the person/party that addresses them, whereas word choices of actors may not necessarily be deliberate. - Advised that scores of virgin texts are not directly comparable to reference texts. Only relative positions of word scores can be interpreted and not raw scores alone. - Virgin text scores tend to have more overlapping, non-discriminating words, resulting in word scores being pulled towards the middle of the scale (clustering effects). Reference text scores tend to be more extreme. However, comparability of virgin and reference texts can be increased by applying the MV transformation that leads to a more even spread in virgin text scores. - Provides increased reliability in texts with 1) words that are shard, 2) where virgin and reference texts are ideologically diametrically opposed. - Provides a relative interpretation of policy positions only (relative to a certain standard/text – virgin text). - Requires technical skills to conduct as using statistical analysis software. - Wordscores analysis is more reliable in long reference texts that contain similar vocabulary as virgin texts. |
| --- | --- |

# Sample and Ethics

| **SAMPLE:**  **Was the sample described in** **detail**?  Yes  No  **Was the sample size justified?**  Yes  No  N/A | **Sampling (who: characteristics; how many; how was sampling done?) If more than one group, was there similarity between groups**?   - Sampling and sample was described in sufficient detail. - Sample chosen based on a previous review of the TPD and included: the tobacco industry, health NGOs, trade unions and representatives from publishing, advertising and retail trade associations. - Sampling conducted via Google searches for publicly available documents in which stakeholders published their positions on tobacco use. - Only English language documents were chosen for analysis: position papers and comments on the TPD consultation paper. - Sample was compiled via Freedom-of-Information requests and comprised of 20 documents from 18 stakeholders. Timeline for document publication: 2010-2013.   **Describe the ethics procedures. Was informed consent obtained**?   - Not required for document analysis. |
| --- | --- |
| **Drop-outs** **were reported?**  Yes  No | **Did any participants drop out from the study? Why? (Were reasons given and were drop-outs handled appropriately**?)   - Not relevant. |

# Results

| **RESULTS:**  **Results were reported in terms of** **statistical significance**?  Yes  No  N/A  Not addressed  **Were the**  **analysis method(s) appropriate**?  Yes  No  Not addressed | **What were the results? Were they statistically significant (i.e., p < 0.05)? If not statistically significant, was study big enough to show an important difference if it should occur? If there were multiple outcomes, was that taken into account for the statistical analysis**?   - Results tabulated and reported in terms of statistical significance. - Established that the EU legislation shifted significantly towards the position held by the tobacco industry lobby groups from policy draft to final policy: from ω=0.52 (95% CI 0.50 to 0.54) to ω=0.40 (95% CI 0.39 to 0.42). The words associated with economy were increasingly more frequent than health language as the policy was developed. - Word frequencies were presented as a percentage of the total number of words in each document. Found that the word ‘health’ comprised a total of 1.71% of all words in health NGO texts but only 0.87% in tobacco industry documents. The usage of word root ‘health’ decreased from 1.50% of total words per document in the initial Commission proposal to 1.21% of total words in the final approved legislation. - Used triangulation by re-confirming validity of results by re-classifying reference texts utilising a different classification model (scaling transformation). - Established that retailers (ω=0.35), trade unions (ω=0.34) and publishers (ω=0.33 and ω=0.40) held policy positions closer to trade unions than that of health NGOs. |
| --- | --- |

# Practical and Theoretical Implications

| **IMPLICATIONS:**  **Were research implications mentioned?**  Yes  No  N/A  **Were practical implications mentioned?**  Yes  No  N/A  **Were theoretical implications mentioned?**  Yes  No  N/A | **Implications for research, practice and theory (what were considered: characteristics?) If comparisons were made, what were they and what were considered between groups**?   - Suggested the use of automated content analysis as an effective and efficient method for evaluation of lobby group activities and health policies and associations between these. - For researchers, recommended the use of automated content analysis methods to boost reliability of analysis results. - Highlighted the importance of Wordscores applications to analyzing the positions of industry groups particularly in large industries and industries were these positions have previously received little attention. |
| --- | --- |

# Conclusions

| **CONCLUSIONS**  **Conclusions were**  **appropriate given**  **study methods and**  **results**  Yes  No | **What did the study conclude? What are the implications of these results for practice? What were the main limitations or biases in the study**?   - Concluded that tobacco industry lobbying impacted the EU tobacco legislation to a significant degree, indicated by a policy shift towards the tobacco industry’s positions. Emphasized the need to address this negative impact by the industry lobby groups in the interest of public health. - Concluded that the Wordscores methodology is effective at analyzing and establishing policy shifts in health legislation. - Recommended that the Wordscores be used with texts with 1) similar vocabulary, and where 2) virgin and reference texts are diametrically opposed.   **Recommendations for future research**   - Future applications of automated content analysis may be useful for detecting potential industry front groups and mobilized third-parties, as Wordscores can be used to identify relative positions of actors. - Recommended to expand the use of the Wordscores method to other health policy fields to gain an understanding of policy positions of various actors that play a role in influencing public policy, including alcohol, food and beverage industries. |
| --- | --- |
